# 5,6-Dihydr­oxy-1,10-phenanthrolin-1-ium chloride dihydrate

**DOI:** 10.1107/S1600536809033777

**Published:** 2009-09-05

**Authors:** Xin-Yong Lin, Sheng-Jiao Tang, Wen-Shi Wu

**Affiliations:** a College of Materials Science and Engineering, Huaqiao University, Xiamen, Fujian 361021, People’s Republic of China

## Abstract

The title compound, C_12_H_9_N_2_O_2_^+^·Cl^−^·2H_2_O, exhibits a layered structure which is stabilized by inter­molecular O—H⋯O, O—H⋯Cl^−^ and N^+^—H⋯Cl^−^ hydrogen bonds, and π–π inter­actions (centroid–centroid distances = 3.654 and 3.583 Å). The distances between the molecules are 3.371 and 3.294 Å.

## Related literature

For a related structure, see: Borel & Bond (2008 ▶).
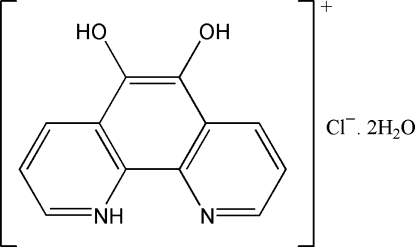


## Experimental

### 

#### Crystal data

C_12_H_9_N_2_O_2_^+^·Cl^−^·2H_2_O
*M* *_r_* = 284.69
Triclinic, 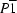

*a* = 7.7627 (1) Å
*b* = 8.6974 (1) Å
*c* = 9.6432 (1) Å
α = 86.116 (1)°
β = 86.859 (1)°
γ = 74.580 (1)°
*V* = 625.73 (1) Å^3^
*Z* = 2
Mo *K*α radiation
μ = 0.32 mm^−1^
*T* = 296 K
0.25 × 0.12 × 0.03 mm

#### Data collection

Bruker P4 diffractometer
Absorption correction: none
9780 measured reflections
2872 independent reflections
2336 reflections with *I* > 2σ(*I*)
*R*_int_ = 0.023

#### Refinement

*R*[*F* ^2^ > 2σ(*F* ^2^)] = 0.036
*wR*(*F* ^2^) = 0.111
*S* = 1.06
2872 reflections
180 parameters
H atoms treated by a mixture of independent and constrained refinement
Δρ_max_ = 0.29 e Å^−3^
Δρ_min_ = −0.22 e Å^−3^

### 

Data collection: *XSCANS* (Bruker, 1999 ▶); cell refinement: *XSCANS* ▶); data reduction: *SHELXTL* (Sheldrick, 2008 ▶); program(s) used to solve structure: *SHELXS97* (Sheldrick, 2008 ▶); program(s) used to refine structure: *SHELXL97* (Sheldrick, 2008 ▶); molecular graphics: *SHELXTL*; software used to prepare material for publication: *SHELXTL*.

## Supplementary Material

Crystal structure: contains datablocks global, I. DOI: 10.1107/S1600536809033777/hg2548sup1.cif

Structure factors: contains datablocks I. DOI: 10.1107/S1600536809033777/hg2548Isup2.hkl

Additional supplementary materials:  crystallographic information; 3D view; checkCIF report

## Figures and Tables

**Table 1:** Hydrogen-bond geometry (Å, °)

*D*—H⋯*A*	*D*—H	H⋯*A*	*D*⋯*A*	*D*—H⋯*A*
O1—H01⋯O4	0.82	1.86	2.6782 (18)	179
O2—H02⋯O3	0.82	1.89	2.6669 (18)	157
O3—H03*B*⋯Cl1	0.81 (3)	2.40 (3)	3.2133 (15)	174 (3)
O3—H03*A*⋯Cl1^i^	0.78 (3)	2.45 (3)	3.2323 (15)	176 (3)
O4—H04*B*⋯Cl1	0.91 (3)	2.33 (3)	3.2185 (14)	165 (3)
O4—H04*A*⋯Cl1^ii^	0.94 (3)	2.30 (3)	3.2216 (14)	168 (3)
N2—H9⋯Cl1^iii^	0.86	2.37	3.1635 (13)	153

Symmetry codes: (i) 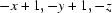; (ii) 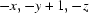; (iii) 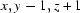.

**Fig. 1.**
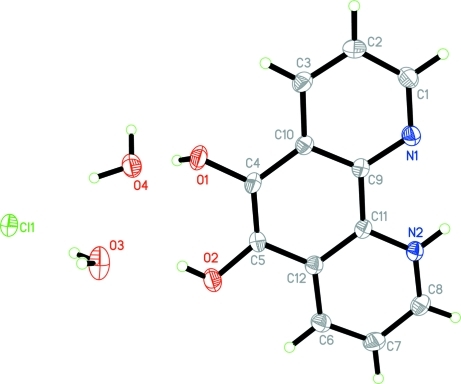
View of the title compound showing the atom-labelling scheme. Displacement ellipsoids are drawn at the 30% probability level. H atoms are represented by circles of arbitrary radius.

**Fig. 2.**
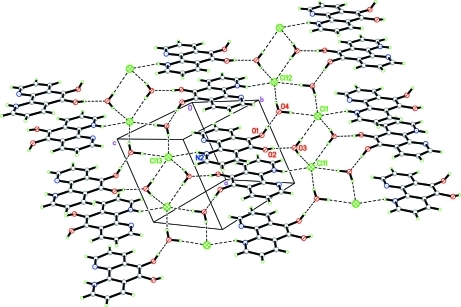
Crystal Packing diagram of the title compound, showing the H-bonded interactions (dashed lines). Cl11, Cl12, Cl13 represent Cl1i, Cl1ii, Cl1iii, respectively.

## supplementary crystallographic information

### Comment

The title compound, [C_12_H_9_N_2_O_2_]Cl.2H_2_O, was obtained unintentionally as the product of an attempted synthesis of a condensation product between 1,10-phenanthroline-5,6-dione and picoloylhydrazide. Compared with a similar compound (5,6-dioxo-1,10-phenanthrolin-1-ium chloride) reported (Borel & Bond, 2008), the Cl—H distance is slightly longer (2.372 vs 2.274Å).

The structure of the title compound is shown in Fig. 1. It exhibits a layered structure which is stabilized by inter-molecular O—H···O, O—H···Cl^-^, N^+^—H···Cl^-^ hydrogen bonds, detailed in Fig. 2 and Table 1, as well as π-π interactions and C—H···O, C—H···Cl^-^ interactions. With Cl^-^ as the connecting point, it occurs two different shape parallelograms made up of O and Cl^-^. The dihedral angle between the two planes, which possess different shapes, is 78.67°. The distances between the layers, which belong to offset face to face, are 3.371 Å and 3.294 Å, reflecting π-π interactions.

### Experimental

1,10-phenanthroline-5,6-dione (300 mg, 1.53 mmol) was dissolved in a mixed solution of 10 ml CH_2_Cl_2_ and 30 ml EtOH when heating with stirring. When all of the compound dissolved, picoloylhydrazide (200 mg, 1.46 mmol) was added and refluxed 8hrs. Then HCl(aq) was added until the pH was 6. Red crystals of the title compound were obtained by slow evaporation of solvent at room temperature. Analysis: Found C 50.45, H 4.82, N 9.71%, calc. for C_12_H_13_ClN_2_O_4_, C 50.63, H 4.60, N 9.84%.

### Refinement

The positions of the O1- , O2- and N2-bound H atoms were placed at fixed positions and refined accord to the riding model.  O3- and O4-bound H atoms were located in a difference Fourier map and refined freely. The C-bound H atoms were included in the riding model approximation with C—H = 0.93 Å and U_iso_ of each H atom = 1.2U_eq_(C).

### Crystal data

**Table:**

C_12_H_9_N_2_O_2_^+^·Cl^−^·2H_2_O	*Z* = 2
*M**_r_* = 284.69	*F*(000) = 296
Triclinic, *P*1	*D*_x_ = 1.511 Mg m^−^^3^
Hall symbol: -P 1	Mo *K*α radiation, λ = 0.71073 Å
*a* = 7.7627 (1) Å	Cell parameters from 2922 reflections
*b* = 8.6974 (1) Å	θ = 2.1–27.7°
*c* = 9.6432 (1) Å	µ = 0.32 mm^−^^1^
α = 86.116 (1)°	*T* = 296 K
β = 86.859 (1)°	Block, red
γ = 74.580 (1)°	0.25 × 0.12 × 0.03 mm
*V* = 625.73 (1) Å^3^	

### Data collection

**Table:**

Bruker P4 diffractometer	2336 reflections with *I* > 2σ(*I*)
Radiation source: fine-focus sealed tube	*R*_int_ = 0.023
graphite	θ_max_ = 27.7°, θ_min_ = 2.1°
Detector resolution: 0 pixels mm^-1^	*h* = −9→9
ω scans	*k* = −11→11
9780 measured reflections	*l* = −12→12
2872 independent reflections	

### Refinement

**Table:**

Refinement on *F*^2^	Primary atom site location: structure-invariant direct methods
Least-squares matrix: full	Secondary atom site location: difference Fourier map
*R*[*F*^2^ > 2σ(*F*^2^)] = 0.036	Hydrogen site location: inferred from neighbouring sites
*wR*(*F*^2^) = 0.111	H atoms treated by a mixture of independent and constrained refinement
*S* = 1.06	*w* = 1/[σ^2^(*F*_o_^2^) + (0.0638*P*)^2^ + 0.0835*P*] where *P* = (*F*_o_^2^ + 2*F*_c_^2^)/3
2872 reflections	(Δ/σ)_max_ < 0.001
180 parameters	Δρ_max_ = 0.29 e Å^−^^3^
0 restraints	Δρ_min_ = −0.22 e Å^−^^3^

### Special details

**Table:**

Geometry. All esds (except the esd in the dihedral angle between two l.s. planes) are estimated using the full covariance matrix. The cell esds are taken into account individually in the estimation of esds in distances, angles and torsion angles; correlations between esds in cell parameters are only used when they are defined by crystal symmetry. An approximate (isotropic) treatment of cell esds is used for estimating esds involving l.s. planes.
Refinement. Refinement of *F*^2^ against ALL reflections. The weighted *R*-factor *wR* and goodness of fit *S* are based on *F*^2^, conventional *R*-factors *R* are based on *F*, with *F* set to zero for negative *F*^2^. The threshold expression of *F*^2^ > σ(*F*^2^) is used only for calculating *R*-factors(gt) *etc*. and is not relevant to the choice of reflections for refinement. *R*-factors based on *F*^2^ are statistically about twice as large as those based on *F*, and *R*- factors based on ALL data will be even larger.

### Fractional atomic coordinates and isotropic or equivalent isotropic displacement parameters (Å^2^)

**Table:**

	*x*	*y*	*z*	*U*_iso_*/*U*_eq_	
Cl1	0.19595 (6)	0.69904 (5)	0.01405 (4)	0.04841 (15)	
O1	0.30334 (16)	0.13935 (13)	0.25607 (10)	0.0444 (3)	
H01	0.2307	0.2235	0.2339	0.067*	
O2	0.37121 (17)	0.34384 (13)	0.45497 (11)	0.0463 (3)	
H02	0.3972	0.3484	0.3715	0.069*	
O3	0.4713 (2)	0.4352 (2)	0.20054 (15)	0.0614 (4)	
H03B	0.404 (3)	0.497 (3)	0.148 (3)	0.078 (8)*	
H03A	0.555 (4)	0.400 (3)	0.152 (3)	0.086 (9)*	
O4	0.06665 (19)	0.41360 (17)	0.18085 (14)	0.0554 (3)	
H04B	0.089 (4)	0.492 (4)	0.121 (3)	0.105 (9)*	
H04A	−0.024 (4)	0.389 (4)	0.133 (3)	0.101 (9)*	
N1	0.18014 (17)	−0.20833 (15)	0.63900 (12)	0.0357 (3)	
N2	0.24864 (16)	−0.00702 (15)	0.82063 (12)	0.0351 (3)	
H9	0.2179	−0.0925	0.8478	0.042*	
C1	0.1482 (2)	−0.30386 (18)	0.54877 (16)	0.0392 (3)	
H1	0.1168	−0.3958	0.5828	0.047*	
C2	0.1591 (2)	−0.27406 (19)	0.40435 (16)	0.0394 (3)	
H2	0.1357	−0.3454	0.3451	0.047*	
C3	0.2039 (2)	−0.14094 (18)	0.35128 (15)	0.0358 (3)	
H3	0.2101	−0.1197	0.2556	0.043*	
C4	0.28852 (19)	0.10836 (17)	0.39662 (13)	0.0321 (3)	
C5	0.32397 (19)	0.20722 (16)	0.48912 (14)	0.0318 (3)	
C6	0.3461 (2)	0.26396 (19)	0.73659 (16)	0.0389 (3)	
H6	0.3798	0.3567	0.7090	0.047*	
C7	0.3317 (2)	0.2208 (2)	0.87618 (16)	0.0456 (4)	
H7	0.3559	0.2838	0.9428	0.055*	
C8	0.2811 (2)	0.0834 (2)	0.91623 (15)	0.0436 (4)	
H8	0.2696	0.0541	1.0102	0.052*	
C9	0.22636 (18)	−0.07646 (16)	0.58613 (13)	0.0298 (3)	
C10	0.24091 (17)	−0.03478 (16)	0.44305 (13)	0.0298 (3)	
C11	0.26175 (17)	0.02913 (16)	0.68159 (13)	0.0299 (3)	
C12	0.31023 (18)	0.16851 (16)	0.63634 (14)	0.0309 (3)	

### Atomic displacement parameters (Å^2^)

**Table:**

	*U*^11^	*U*^22^	*U*^33^	*U*^12^	*U*^13^	*U*^23^
Cl1	0.0659 (3)	0.0498 (3)	0.0337 (2)	−0.0246 (2)	−0.00378 (17)	0.00808 (16)
O1	0.0661 (8)	0.0399 (6)	0.0248 (5)	−0.0116 (5)	0.0009 (5)	0.0034 (4)
O2	0.0697 (8)	0.0360 (6)	0.0367 (6)	−0.0223 (5)	0.0041 (5)	0.0027 (5)
O3	0.0576 (9)	0.0664 (9)	0.0517 (8)	−0.0087 (7)	0.0078 (7)	0.0172 (7)
O4	0.0623 (8)	0.0580 (8)	0.0512 (7)	−0.0280 (6)	−0.0123 (6)	0.0164 (6)
N1	0.0414 (7)	0.0340 (6)	0.0329 (6)	−0.0132 (5)	0.0009 (5)	0.0003 (5)
N2	0.0447 (7)	0.0361 (7)	0.0263 (6)	−0.0146 (5)	0.0005 (5)	0.0019 (5)
C1	0.0455 (9)	0.0327 (8)	0.0410 (8)	−0.0138 (6)	0.0024 (6)	−0.0018 (6)
C2	0.0442 (9)	0.0366 (8)	0.0392 (8)	−0.0115 (6)	−0.0011 (6)	−0.0112 (6)
C3	0.0396 (8)	0.0382 (8)	0.0283 (7)	−0.0074 (6)	−0.0011 (5)	−0.0036 (6)
C4	0.0369 (7)	0.0333 (7)	0.0235 (6)	−0.0061 (5)	0.0002 (5)	0.0021 (5)
C5	0.0347 (7)	0.0279 (7)	0.0318 (7)	−0.0078 (5)	0.0000 (5)	0.0037 (5)
C6	0.0454 (9)	0.0364 (8)	0.0380 (8)	−0.0160 (6)	−0.0007 (6)	−0.0045 (6)
C7	0.0586 (10)	0.0495 (10)	0.0343 (8)	−0.0218 (8)	−0.0033 (7)	−0.0102 (7)
C8	0.0556 (10)	0.0525 (10)	0.0251 (7)	−0.0183 (7)	−0.0004 (6)	−0.0029 (6)
C9	0.0306 (7)	0.0306 (7)	0.0273 (6)	−0.0070 (5)	−0.0004 (5)	0.0002 (5)
C10	0.0297 (7)	0.0313 (7)	0.0271 (6)	−0.0056 (5)	−0.0008 (5)	−0.0019 (5)
C11	0.0297 (7)	0.0335 (7)	0.0256 (6)	−0.0072 (5)	−0.0012 (5)	0.0003 (5)
C12	0.0305 (7)	0.0323 (7)	0.0296 (7)	−0.0081 (5)	−0.0005 (5)	−0.0006 (5)

### Geometric parameters (Å, °)

**Table:**

O1—C4	1.3685 (16)	C2—H2	0.9300
O1—H01	0.8200	C3—C10	1.414 (2)
O2—C5	1.3491 (18)	C3—H3	0.9300
O2—H02	0.8200	C4—C5	1.367 (2)
O3—H03B	0.81 (3)	C4—C10	1.428 (2)
O3—H03A	0.78 (3)	C5—C12	1.4415 (18)
O4—H04B	0.91 (3)	C6—C7	1.380 (2)
O4—H04A	0.94 (3)	C6—C12	1.401 (2)
N1—C1	1.3192 (19)	C6—H6	0.9300
N1—C9	1.3504 (18)	C7—C8	1.379 (2)
N2—C8	1.327 (2)	C7—H7	0.9300
N2—C11	1.3608 (17)	C8—H8	0.9300
N2—H9	0.8600	C9—C10	1.4088 (18)
C1—C2	1.401 (2)	C9—C11	1.4293 (19)
C1—H1	0.9300	C11—C12	1.3980 (19)
C2—C3	1.355 (2)		
			
C4—O1—H01	109.5	C4—C5—C12	119.73 (13)
C5—O2—H02	109.5	C7—C6—C12	120.17 (14)
H03B—O3—H03A	103 (2)	C7—C6—H6	119.9
H04B—O4—H04A	99 (2)	C12—C6—H6	119.9
C1—N1—C9	116.77 (12)	C8—C7—C6	119.53 (14)
C8—N2—C11	123.10 (13)	C8—C7—H7	120.2
C8—N2—H9	118.4	C6—C7—H7	120.2
C11—N2—H9	118.4	N2—C8—C7	119.93 (14)
N1—C1—C2	123.39 (14)	N2—C8—H8	120.0
N1—C1—H1	118.3	C7—C8—H8	120.0
C2—C1—H1	118.3	N1—C9—C10	124.54 (12)
C3—C2—C1	119.85 (14)	N1—C9—C11	117.94 (12)
C3—C2—H2	120.1	C10—C9—C11	117.52 (12)
C1—C2—H2	120.1	C9—C10—C3	116.20 (13)
C2—C3—C10	119.24 (13)	C9—C10—C4	120.66 (12)
C2—C3—H3	120.4	C3—C10—C4	123.13 (12)
C10—C3—H3	120.4	N2—C11—C12	118.90 (12)
C5—C4—O1	121.50 (13)	N2—C11—C9	119.19 (12)
C5—C4—C10	121.14 (12)	C12—C11—C9	121.91 (12)
O1—C4—C10	117.32 (12)	C11—C12—C6	118.36 (13)
O2—C5—C4	125.29 (12)	C11—C12—C5	119.02 (12)
O2—C5—C12	114.97 (12)	C6—C12—C5	122.62 (13)
			
C9—N1—C1—C2	−0.4 (2)	O1—C4—C10—C9	178.79 (12)
N1—C1—C2—C3	−0.2 (2)	C5—C4—C10—C3	−179.72 (13)
C1—C2—C3—C10	0.7 (2)	O1—C4—C10—C3	−2.0 (2)
O1—C4—C5—O2	1.8 (2)	C8—N2—C11—C12	−0.5 (2)
C10—C4—C5—O2	179.45 (13)	C8—N2—C11—C9	179.24 (13)
O1—C4—C5—C12	−178.75 (12)	N1—C9—C11—N2	0.33 (19)
C10—C4—C5—C12	−1.1 (2)	C10—C9—C11—N2	179.86 (12)
C12—C6—C7—C8	−0.3 (3)	N1—C9—C11—C12	−179.92 (12)
C11—N2—C8—C7	−0.4 (2)	C10—C9—C11—C12	−0.4 (2)
C6—C7—C8—N2	0.8 (3)	N2—C11—C12—C6	1.0 (2)
C1—N1—C9—C10	0.5 (2)	C9—C11—C12—C6	−178.75 (13)
C1—N1—C9—C11	−179.99 (13)	N2—C11—C12—C5	−179.91 (12)
N1—C9—C10—C3	−0.1 (2)	C9—C11—C12—C5	0.3 (2)
C11—C9—C10—C3	−179.56 (12)	C7—C6—C12—C11	−0.6 (2)
N1—C9—C10—C4	179.20 (12)	C7—C6—C12—C5	−179.68 (14)
C11—C9—C10—C4	−0.29 (19)	O2—C5—C12—C11	179.92 (12)
C2—C3—C10—C9	−0.5 (2)	C4—C5—C12—C11	0.4 (2)
C2—C3—C10—C4	−179.81 (14)	O2—C5—C12—C6	−1.0 (2)
C5—C4—C10—C9	1.1 (2)	C4—C5—C12—C6	179.47 (13)

### Hydrogen-bond geometry (Å, °)

**Table:**

*D*—H···*A*	*D*—H	H···*A*	*D*···*A*	*D*—H···*A*
O1—H01···O4	0.82	1.86	2.6782 (18)	179
O2—H02···O3	0.82	1.89	2.6669 (18)	157
O3—H03B···Cl1	0.81 (3)	2.40 (3)	3.2133 (15)	174 (3)
O3—H03A···Cl1^i^	0.78 (3)	2.45 (3)	3.2323 (15)	176 (3)
O4—H04B···Cl1	0.91 (3)	2.33 (3)	3.2185 (14)	165 (3)
O4—H04A···Cl1^ii^	0.94 (3)	2.30 (3)	3.2216 (14)	168 (3)
N2—H9···Cl1^iii^	0.86	2.37	3.1635 (13)	153

Symmetry codes: (i) −*x*+1, −*y*+1, −*z*; (ii) −*x*, −*y*+1, −*z*; (iii) *x*, *y*−1, *z*+1.
